# A Novel Machine-Learning Based Method for Resolving Secondary Structure Topology in Medium-Resolution Cryo-EM Density Maps

**DOI:** 10.3390/ijms27104388

**Published:** 2026-05-14

**Authors:** Bahareh Behkamal, Mohammad Parsa Etemadheravi, Ali Mahmoodjanloo, Amin Mansoori, Mahmoud Naghibzadeh, Kamal Al Nasr, Mohammad Reza Saberi

**Affiliations:** 1 Medicinal Chemistry Department, School of Pharmacy, Mashhad University of Medical Sciences, Mashhad 9177899191, Iran; b.behkamal@alumni.um.ac.ir; 2 Department of Computer Engineering, Faculty of Engineering, Ferdowsi University of Mashhad, P.O. Box 1159, Mashhad 9177948974, Iran; etemadheravi.mohammadparsa@mail.um.ac.ir (M.P.E.); naghibzadeh@um.ac.ir (M.N.); 3 Department of Applied Mathematics, Faculty of Mathematical Sciences, Ferdowsi University of Mashhad, P.O. Box 1159, Mashhad 917751159, Iran; ali.mahmoodjanloo@alumni.um.ac.ir (A.M.); aminmansoori@um.ac.ir (A.M.); 4 Mathematical Biology Research Laboratory (MBRL), Faculty of Mathematical Sciences, Ferdowsi University of Mashhad, P.O. Box 1159, Mashhad 917751159, Iran; 5 Department of Computer Science, Tennessee State University, Nashville, TN 37209, USA; 6 Bioinformatics Research Group, Mashhad University of Medical Sciences, Mashhad 9177899191, Iran

**Keywords:** cryo-electron microscopy, protein secondary structure, topology determination, medium resolution, modeling, classification, machine learning, classification

## Abstract

Medium-resolution cryo-electron microscopy (cryo-EM) density maps preserve substantial information about protein secondary-structure organization; however, accurately recovering the topology and connectivity of α-helices and β-strands remains challenging due to noise, structural heterogeneity, and the intrinsic resolution limitations that obscure residue-level detail. Topology determination is a key intermediate step toward building atomic protein models from medium-resolution cryo-EM density maps. It requires identifying the correct correspondence and orientation between secondary-structure elements (SSEs), i.e., α-helices and β-strands, predicted from the amino-acid sequence and those detected in the three dimensional (3D) density map. Despite significant advances in cryo-EM reconstruction and molecular modelling, this correspondence problem remains a challenging task, particularly in the presence of noisy density maps and in large, topologically complex α/β proteins. To address this issue, we propose a fully automated, classification-based framework that infers protein secondary-structure topology directly from medium-resolution cryo-EM density maps. Specifically, we cast topology determination as a supervised classification problem in three-dimensional space, leveraging geometric learning on model-derived Cα coordinate representations to establish SSE correspondences, and a Dynamic Time Warping (DTW)-based procedure to resolve density-stick directionality. Validation on a benchmark of 38 proteins spanning both simulated and experimental cryo-EM maps and covering diverse fold classes (α, β, and α/β) demonstrates strong and consistent performance. Among the evaluated predictors, the Voronoi (1-NN) classifier achieves the highest average correspondence quality, with a mean F1-score of 96.82% across the full benchmark. The framework also scales to large, topologically dense targets containing up to 65 secondary-structure elements while preserving very fast correspondence inference (<3 ms), offering a substantial improvement over prior baselines in both accuracy and computational cost. Overall, the classification-driven strategy provides reliable SSE-to-density matching and, when coupled with DTW-based direction selection, yields stronger topology constraints that directly support model building and refinement from medium-resolution cryo-EM reconstructions, while remaining easy to integrate into existing structural interpretation pipelines.

## 1. Introduction

**Figure 1:**
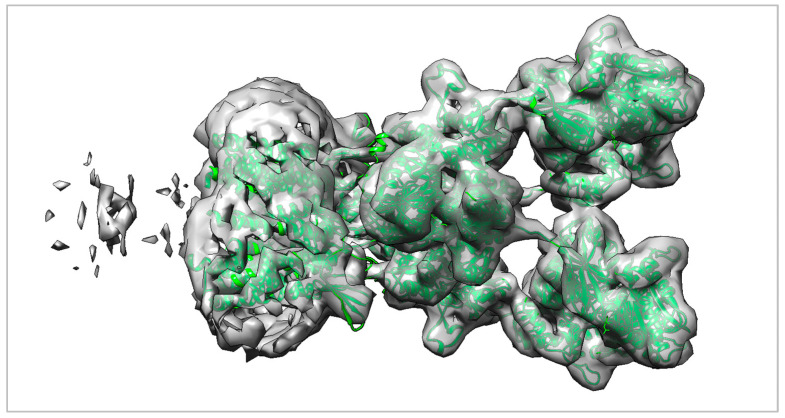
Representative superimposition of the fitted atomic model and medium-resolution cryo-EM density map for PDB 5KBU/EMDB EMD-8231. The cryo-EM density map is shown as a semi-transparent grey surface, while the fitted atomic model is displayed in a contrasting green ribbon representation. At medium resolution, the global molecular envelope and major secondary-structure elements, including α-helices and β-strands, can be visually recognized; however, individual side chains and residue-level details are not reliably resolved.

Accurate determination of protein secondary structures is fundamental to structural biology, as it underpins our understanding of protein function, dynamics, and interactions [1,2]. Single-particle cryogen electron microscopy (cryo-EM) has emerged as a powerful technique for resolving three-dimensional (3D) protein structures at increasingly high resolution, enabling the visualization of intricate molecular architectures without the need for crystallization [3,4,5,6,7,8,9]. Despite substantial progress in cryo-EM reconstruction, medium-resolution maps (4–6 Å) still pose major challenges for accurate model building [10,11,12,13,14,15]. Local variations in density, conformational heterogeneity, and low signal-to-noise ratios hinder the reliable assignment of amino acid types and secondary-structure elements and typically require considerable manual intervention and expert knowledge [16]. The computational analysis of cryo-EM density maps has progressed through several distinct stages [17,18,19]. Early methods relied primarily on template-based strategies and manual feature engineering, which achieved only limited success in automating the structure determination pipeline [20,21]. Classical machine-learning techniques, particularly Support Vector Machines (SVMs), showed promise for pattern-recognition tasks relevant to protein structure analysis [22]. The pioneering study by Si et al. [23] established the first SVM-based baseline for SSE identification directly from cryo-EM maps; however, its performance was constrained by limited exploitation of 3D spatial context and a strong dependence on hand-crafted features. Deep learning has introduced transformative capabilities to cryo-EM density map analysis [24,25,26]. Three-dimensional convolutional neural networks (3D CNNs) emerged as powerful tools for capturing volumetric context, with early implementations in 2016 [27] highlighting the importance of 3D feature learning for accurate secondary-structure identification. Subsequent architectures, including 3D Inception-style networks (2021) [28], further enhanced performance through multi-scale feature extraction. More recent segmentation frameworks, such as EMNUSS (3D nested U-Net, 2023) [29] and cryoSSESeg (3D U-Net, 2024) [30], have reported high accuracy, particularly for the challenging task of β-sheet detection. Despite these advances, deep-learning approaches present several practical limitations: (i) substantial computational demands, which restrict their accessibility for many research groups [25,31]; (ii) the need for large, well-curated training datasets and careful hyperparameter tuning [32]; (iii) limited interpretability of the learned features [33]; and (iv) potential overfitting to specific map characteristics or reconstruction artefacts [34]. Accurate determination of protein secondary structures from medium-resolution cryo-EM maps requires solving two tightly coupled problems. First, the SSE correspondence problem consists of establishing the correct assignment between secondary-structure elements (SSEs) predicted from the protein sequence and those observed in the cryo-EM density map [35,36,37]. This is essentially a combinatorial matching task, complicated by incomplete, noisy, or fragmented density [38,39,40]. Second, the SSE orientation problem concerns determining the N- to C-terminal direction of each matched SSE, which is crucial for backbone tracing and subsequent atomic model building. Previous graph-based approaches [1,3,38] have attempted to tackle these challenges using skeleton-derived features and length information. However, cryo-EM skeletons are often unstable in the presence of noise and reconstruction artefacts, and SSE length alone provides limited discriminative power when multiple elements exhibit similar dimensions [1,3,41,42]. For additional clarity and to illustrate the practical context of the topology-determination problem, Figure 1 presents an example of a medium-resolution cryo-EM density map superimposed with its fitted atomic model (PDB ID: 5KBU; EMDB ID: EMD-8231). At medium resolution, α-helices and β-sheets appear as elongated or planar density regions, yet individual residues are not resolved. Correctly identifying and matching these secondary-structure elements between the sequence-derived model and the cryo-EM density is therefore essential for stabilizing backbone tracing and preventing topological inconsistencies during model building.

To further position the proposed framework relative to representative deep-learning approaches, we compare commonly used methods for cryo-EM map interpretation. For example, 3D CNN-based secondary-structure detection [28], EMNUSS [29], cryoSSESeg [30], DeepMM [43], CR-I-TASSER [44], and cascaded CNN-based backbone prediction [45] have demonstrated the value of deep learning for volumetric segmentation, secondary-structure annotation, backbone tracing, and automated model building from cryo-EM density maps. These methods provide important upstream or downstream capabilities in the cryo-EM interpretation pipeline. However, these deep-learning methods do not directly solve the specific correspondence task addressed in this study unless they are modified to output SSE-to-SSE correspondence labels and are evaluated under the same incomplete-matching protocol. The present work focuses on the downstream topology-determination stage, namely assigning correspondence and direction between sequence/model-derived SSEs and density-map-derived SSEs.

In this study, we propose a classification-based matching framework to infer the correct correspondence between sequence-derived secondary-structure elements (SSEs-S) and density-derived SSEs extracted from cryo-EM maps (SSEs-V). The method operates directly on 3D Cα coordinate sequences, enabling supervised predictors to learn intrinsic geometric signatures of helices and strands from their native spatial patterns, rather than relying on abstracted encodings, density skeletons, or simple length-based heuristics that are often brittle under noise, missing density, and segmentation errors at medium resolution. The central idea is to recast SSE mapping as a 3D classification problem: we represent each candidate SSE pair in Cartesian space using a consistent set of geometric features and assign it a correspondence label, yielding training and test sets compatible with standard supervised learning and allowing correspondence estimation to be learned from data in a unified pipeline. Figure 1 illustrates the practical context of the proposed topology-determination framework, in which secondary-structure correspondence and orientation assignment are required to support downstream model building and refinement.

We systematically evaluate multiple classifiers, including SVMs (linear and RBF kernels), Random Forests, and k-Nearest Neighbors (Voronoi), and identify reliable configurations for candidate ranking. Finally, to produce a unique and globally consistent SSE assignment, we further introduce a Dynamic Time Warping (DTW)-based orientation module that determines SSE directions and resolves ambiguities among competing matches. Our results show that all evaluated machine-learning classifiers deliver consistently high accuracy for SSE correspondence. The Voronoi predictor achieves the best overall performance (96.82%), followed closely by SVM-linear (96.22%), Random Forest (96.18%), and SVM-RBF (95.99%), indicating that the proposed learning formulation is robust to the choice of classifier and scales reliably to complex proteins with up to 65 SSEs. When analyzed by SSE type, the framework attains 96.92% accuracy for α-helices and 82.65% for β-strands, reflecting the greater ambiguity typically associated with strand-like density at medium resolution. Importantly, this accuracy is achieved with practical runtimes under 3 ms, offering a favourable efficiency–accuracy trade-off and matching or substantially exceeding more computationally demanding approaches. Its rapid and reliable predictions can augment or replace feature-prediction stages in downstream modelling pipelines such as DeepMM [43], CR-I-TASSER [44], and cascaded CNN backbone tracers [45], thereby accelerating de novo structure determination workflows.

Additional recent advances further illustrate the rapid development of AI-assisted cryo-EM interpretation. Haruspex [46], Emap2sec+ [47], and DeepSSETracer [48] provide representative deep-learning tools for cryo-EM density-map annotation and secondary-structure segmentation. Automated and hybrid model-building frameworks, including EMBuild [49] and DEMO-EM2 [50], combine predicted structural models, density-map fitting, and iterative assembly strategies for cryo-EM model construction. Large labelled resources such as Cryo2StructData [51] also support the training and benchmarking of AI-based cryo-EM modelling methods. These studies clarify that the proposed framework addresses a distinct but complementary downstream task: resolving SSE-to-SSE correspondence and orientation after secondary-structure elements have been identified.

The remainder of this paper is organized as follows. Section 2 reports comprehensive results, comparing multiple classifiers for SSE correspondence and direction resolution. Section 3 discusses the implications and limitations of the proposed approach and outlines directions for future work. Section 4 presents the proposed methodology, including structural model construction, voxel extraction from the 3D model and cryo-EM volume, supervised classification strategies, and SSE orientation determination. Section 5 concludes the work.

## 2. Results

This section reports experimental validation of the proposed classification-based framework for determining secondary-structure topology from medium-resolution cryo-EM density maps. We first describe the adopted benchmark dataset and evaluation protocol, then present correspondence and orientation results across protein classes and levels of structural complexity, including a direct comparison against the LPTD baseline [3].

### 2.1. Benchmark Dataset

To enable a direct and reproducible comparison with prior topology/correspondence studies, we adopt a consolidated benchmark assembled from the datasets used in our three earlier works [1,3,38] and summarized in the accompanying Table 1. Overall, the benchmark comprises 38 unique proteins (PDB IDs) spanning a wide range of sizes and structural complexity. The sequence lengths cover approximately 96–1703 amino acids, and the structural content includes both α-helical and α/β architectures. Importantly, the benchmark includes challenging large proteins with up to 65 secondary-structure elements (SSEs), enabling scalability in dense correspondence settings. The benchmark contains both simulated and experimental cryo-EM density maps to capture complementary sources of variability. The simulated maps were generated from known atomic structures (PDB) at medium resolution, mainly 10 Å, using established map-simulation procedures adopted in previous topology-determination studies, including Chimera “molmap” for the α/β benchmark and EMAN-based simulation for the α-protein benchmark. These procedures apply resolution-dependent smoothing/degradation when converting high-resolution atomic coordinates into volumetric density maps, thereby mimicking the loss of high-frequency structural information that occurs in medium-resolution cryo-EM. Consequently, individual residues and side-chain details are no longer reliably resolved, while secondary-structure-level features remain detectable. We retained these benchmark maps without introducing additional artificial filtering or noise in order to enable a direct, like-for-like comparison with previously published baseline methods evaluated on the same datasets, including LPTD and related topology-determination approaches. In addition to the simulated maps, experimental density maps were retrieved from the Electron Microscopy Data Bank (EMDB), paired with their fitted atomic structures/homologues, and used to evaluate the method under more realistic reconstruction conditions. Across the adopted benchmarks, the experimental map resolutions span approximately 3.7–8.9 Å for the α/β set and 6–10 Å for the α-protein set, thereby covering the typical medium-resolution range where SSEs are detectable but full atomic tracing remains non-trivial.

### 2.2. Performance Measurements

This study evaluates the robustness of the proposed method under noise and uncertainty in reconstructed cryo-EM data. In practice, detection and segmentation errors may cause discrepancies between SSEs-S and SSEs-V. As a result, the number of extracted SSEs-V is often different from the number of SSEs-S, and a complete one-to-one correspondence cannot always be established. Therefore, performance must be assessed in the presence of incomplete matching. To this end, we partition the instances into two classes including matched classes which are SSEs-S that have a corresponding SSEs-V (i.e., predicted matched pairs) and unmatched class SSEs-S with no corresponding SSEs-V (i.e., predicted as unmatched). Based on these classes, we compute Precision, Recall, and F1-measure rate as follows:

(1) $$Precision=TP/\left(TP+FP\right)*100,$$

(2) $$Recall=TP/\left(TP+FN\right)*100,$$

(3) $$F1\text{-}measure=\left(2\times Precision\times Recall\right)/\left(Precision+Recall\right)*100,$$

Here, $TP\left(TruePositive\right)$ is the number of correctly identified matched pairs; $FP\left(FalsePositive\right)$ is the number of incorrectly assigned matched pairs (wrong correspondences); and $FN\left(FalseNegative\right)$ is the number of true matched pairs that were missed or rejected by the method. In addition, $TN\left(TrueNegative\right)$ denotes the number of correctly identified unmatched cases (i.e., SSEs-S correctly left without correspondence).

### 2.3. Overall Correspondence Performance Across Classifiers

**Figure 2:**
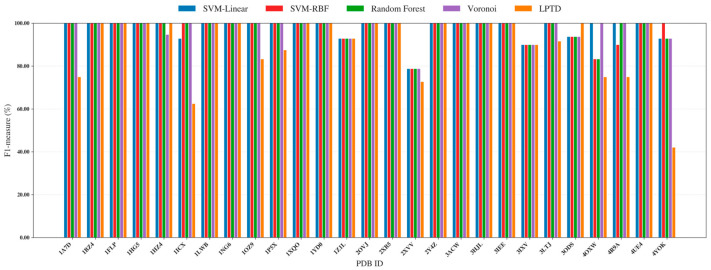
Per-protein F1-measure (%) for SSE correspondence on the simulated subset of benchmark proteins, comparing the proposed supervised classifiers (SVM-linear, SVM-RBF, Random Forest, and Voronoi) against the LPTD baseline.

**Figure 3:**
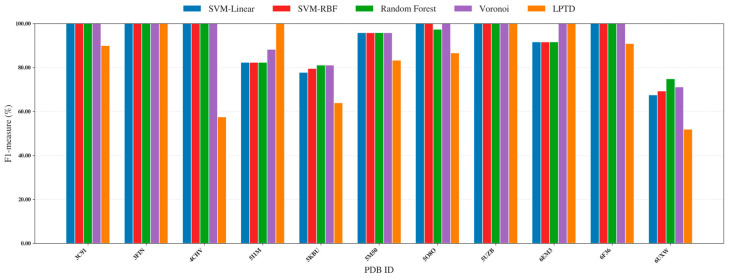
Per-protein F1-measure (%) for SSE correspondence on the experimental subset of benchmark proteins, comparing the proposed supervised classifiers (SVM-linear, SVM-RBF, Random Forest, and Voronoi) against the LPTD baseline.

We evaluated four correspondence predictors within the same unified learning pipeline, including SVM-linear, SVM-RBF, Random Forest, and Voronoi, and benchmarked them against the LPTD baseline previously reported in [3]. All methods operate on an identical 3D representation (Figure 2 and Figure 3): each model-derived SSE (SSEs-S) is encoded as a Cα coordinate sequence, and each map-derived density stick (SSEs-V) is encoded as a stick–axis coordinate sequence. Hyperparameters were selected via cross-validated search on the training set and then fixed and applied consistently across all 38 benchmark proteins to ensure a fair, like-for-like comparison. The per-protein F1 results (Figure 2 and Figure 3) are reported separately for simulated and experimental datasets. Across both subsets, the supervised predictors achieve consistently high correspondence quality over diverse targets, frequently approaching near-perfect F1 scores. In contrast, LPTD shows noticeably larger variability, with more pronounced performance drops on several challenging protein—particularly in the experimental subset, where density-stick extraction and segmentation errors are typically more frequent. Overall, the proposed formulation remains robust across a wide range of protein sizes and fold classes (α, β, and α/β). This robustness is especially evident for difficult α/β targets, where the candidate correspondence space expands rapidly and errors in stick segmentation can propagate, underscoring the benefit of casting correspondence estimation as a supervised classification problem.

**Figure 4:**
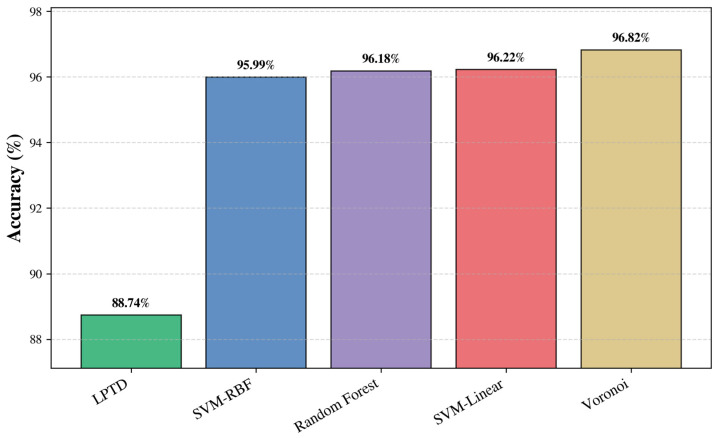
Average SSE correspondence accuracy (%) across the 38-protein benchmark. The proposed supervised classifiers (SVM-RBF, Random Forest, SVM-linear, and Voronoi) are compared against the LPTD baseline; bar labels report the mean accuracy achieved by each method (highest: Voronoi at 96.82%).

As shown in Figure 4, the average performance over the full 38-protein benchmark indicates that Voronoi attains the highest mean correspondence score (96.82%), followed closely by SVM-linear (96.22%), Random Forest (96.18%), and SVM-RBF (95.99%), while the LPTD baseline achieves 88.74%. Overall, the proposed classification-based formulation yields an absolute gain of approximately 7–8 percentage points in mean correspondence quality relative to the baseline on this consolidated dataset.

**Figure 5:**
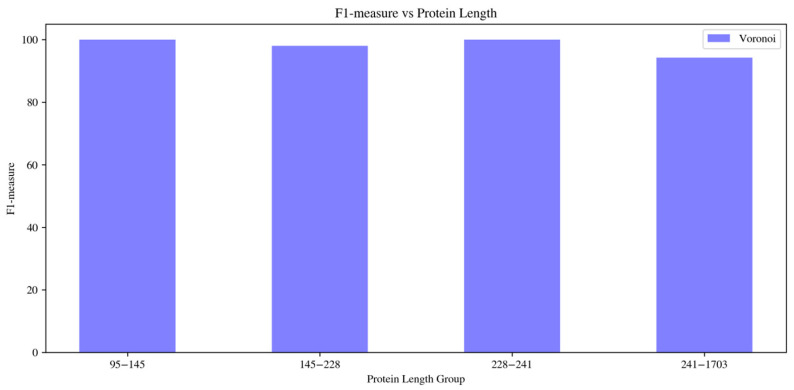
Average F1-measure of the Voronoi classifier across different protein sequence-length ranges. The results show that the Voronoi-based correspondence predictor maintains high performance across most protein-size groups, with a moderate decrease in the largest group due to increased structural complexity and a larger SSE correspondence search space.

To further investigate whether correspondence accuracy depends on protein size, we grouped the benchmark proteins according to their sequence-length ranges and computed the average F1-measure for each evaluated predictor within each group. As shown in Figure 5, the Voronoi classifier maintains very high performance across most length ranges, achieving 100.00% average F1-measure in the 96–145 and 228–341 residue groups, 98.10% in the 145–228 residue group, and 89.65% in the largest 341–1703 residue group. The moderate decrease observed for the largest proteins is expected, because larger targets usually contain more secondary-structure elements and therefore create a larger and more ambiguous correspondence search space. Nevertheless, the Voronoi-based predictor remains robust even in this more challenging group.

**Figure 6:**
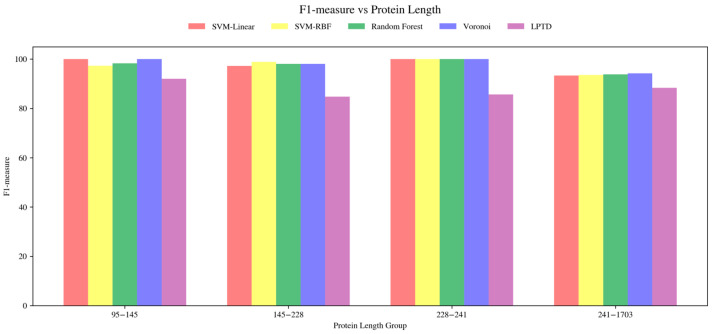
Average F1-measure of the evaluated SSE correspondence predictors across different protein sequence-length ranges. The benchmark proteins were grouped according to sequence length, and the mean F1-measure was computed for each method within each group. The proposed supervised classifiers maintain high correspondence accuracy across all protein-size ranges and generally outperform the LPTD baseline, particularly in larger and more structurally complex proteins.

We also compared all evaluated predictors across the same protein-length groups. As shown in Figure 6, the proposed supervised classifiers, including SVM-linear, SVM-RBF, Random Forest, and Voronoi, maintain consistently high F1-measure values across all length ranges and generally outperform the LPTD baseline. In the largest length group, 341–1703 residues, where the correspondence task is most difficult, the supervised classifiers achieve average F1-measure values of approximately 88.9–89.7%, whereas LPTD reaches 82.1%. These results indicate that the proposed classification-based correspondence formulation is more robust than the LPTD baseline under increasing protein size and structural complexity.

To assess whether the observed differences in mean correspondence accuracy are statistically significant, we conducted pairwise comparisons using paired *t*-tests on the per-protein F1-scores across the 38-protein benchmark. This paired design is appropriate because all predictors were evaluated on the same set of protein targets, allowing method-to-method differences to be assessed while accounting for protein-specific variability. The results are reported in Table 2. The pairwise comparisons show that the performance differences among the proposed supervised classifiers, Voronoi, SVM-linear, SVM-RBF, and Random Forest, are not statistically significant at the 0.05 level, despite small variations in their average F1-scores. By contrast, all proposed supervised classifiers demonstrate statistically significant improvements over the LPTD baseline, with *p*-values below 0.01.

### 2.4. Helix Correspondence Accuracy

**Figure 7:**
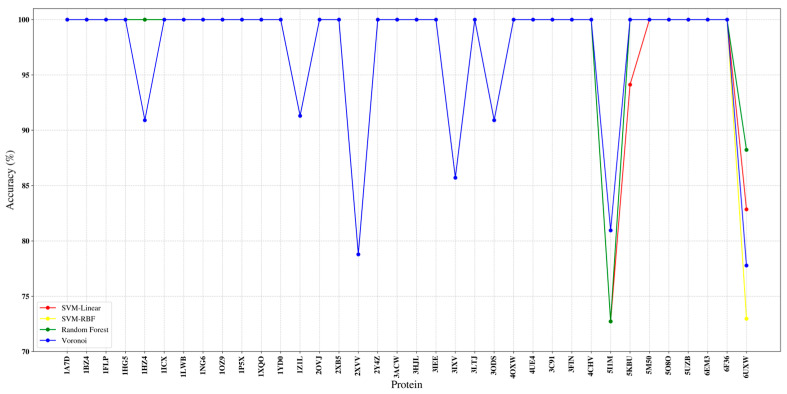
Per-protein α-helix correspondence accuracy on the 38-protein benchmark for the evaluated supervised classifiers (SVM-linear, SVM-RBF, Random Forest, and Voronoi).

To better isolate behaviour by SSE type, we report per-protein correspondence accuracy for α-helices (Figure 7). Across most targets, all four classifiers achieve near-ceiling accuracy, with many proteins clustered in the 0.95–1.00 range, indicating that helix geometry in medium-resolution maps is generally distinctive and can be learned reliably from the 3D coordinate representation. Nevertheless, Figure 7 also highlights a small number of challenging cases that introduce visible drops for all methods (e.g., proteins with pronounced density fragmentation, strong heterogeneity, or severe model-map inconsistency), thereby providing informative stress tests for robustness. In particular, the concurrent drop across classifiers for specific outliers suggests that the dominant error source is often upstream (SSE detection quality, missing sticks, or ambiguous helix-like density) rather than classifier capacity alone.

### 2.5. Strand Correspondence Accuracy

**Figure 8:**
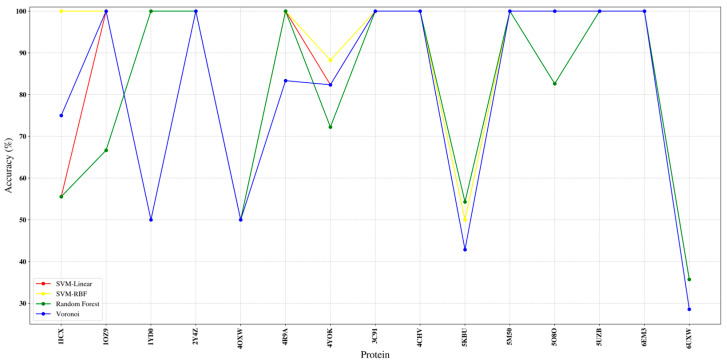
β-strand correspondence accuracy (per protein) for the evaluated supervised classifiers (SVM-linear, SVM-RBF, Random Forest, and Voronoi) on the strand-containing subset of the 38-protein benchmark; each curve reports the fraction of correctly matched strand SSEs between model-derived SSEs-S and map-derived SSEs-V for a given target.

β-strands are typically more difficult than helices in medium-resolution cryo-EM due to weaker tubular signatures and frequent merging into sheet-like densities. The strand-matching task exhibits larger variance than the helix case, reflecting the well-known challenges of β-structure detection and segmentation in medium-resolution maps (shorter elements, weaker/fragmented density, and higher sensitivity to missing or merged sticks). Figure 8 reports the per-protein β-strand correspondence accuracy for the subset of benchmark proteins that contain strands (i.e., α/β proteins), comparing SVM-linear, SVM-RBF, Random Forest, and Voronoi under the same feature construction and evaluation protocol. Across most targets, all four predictors reach high accuracy (typically ≥ 0.85), with Voronoi and SVM-RBF frequently achieving the strongest or near-strongest performance, while Random Forest remains competitive but shows occasional drops.

### 2.6. Runtime and Computational Efficiency

**Figure 9:**
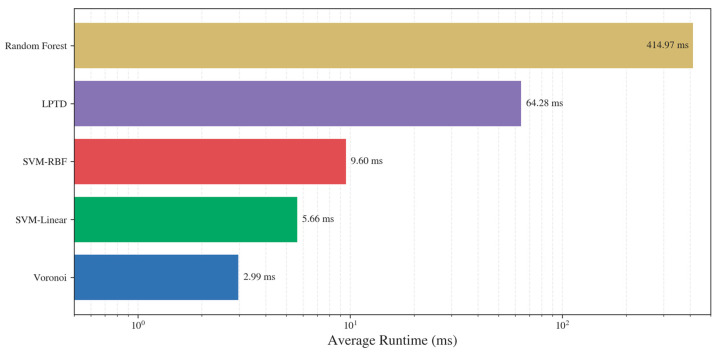
Average runtime comparison of SSE correspondence methods on the 38-protein benchmark. The proposed classifiers (Voronoi, SVM-linear, SVM-RBF, and Random Forest) are contrasted with the prior LPTD baseline, highlighting the substantially lower computational cost of Voronoi and SVM variants.

To evaluate practical usability, we compared the average end-to-end runtime of the competing correspondence predictors on a Dell Latitude E6440 (2.7 GHz quad-core Intel Core i5, 8 GB RAM) (Figure 9). Runtimes are reported in milliseconds to capture the wide variability across methods. The results show that the proposed Voronoi is the most efficient predictor, with an average runtime on the order of a few milliseconds per protein. SVM-linear remains lightweight (single-digit milliseconds), while SVM-RBF incurs a modest additional cost (around one order of magnitude higher than Voronoi) due to non-linear kernel evaluation. In contrast, LPTD and especially Random Forest are substantially slower, reaching tens to hundreds of milliseconds on average, consistent with the overhead of more complex matching machinery (LPTD) and the evaluation of many decision trees (Random Forest). Overall, Figure 9 highlights that the proposed classification formulation not only improves correspondence quality but also provides a favourable speed–accuracy trade-off, enabling rapid topology determination suitable for integration into high-throughput cryo-EM interpretation pipelines.

**Figure 10:**
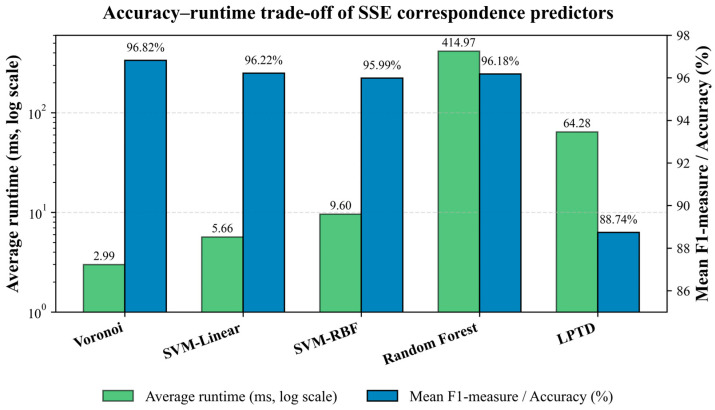
Accuracy–runtime comparison of the evaluated SSE correspondence predictors. The left axis reports the mean correspondence accuracy, while the right axis reports the average runtime in milliseconds using a logarithmic scale. Voronoi achieves the best overall trade-off, combining the highest average accuracy (96.82%) with the lowest runtime (2.99 ms).

To further clarify the trade-off between predictive accuracy and computational efficiency, we added a direct accuracy–runtime comparison for all evaluated SSE predictors. As shown in Figure 10, the Voronoi classifier provides the most favourable overall balance, achieving the highest mean correspondence accuracy (96.82%) while also requiring the lowest average runtime (2.99 ms). SVM-linear also represents a highly efficient practical alternative, with a comparable mean accuracy of 96.22% and an average runtime of 5.66 ms. SVM-RBF achieves similar accuracy (95.99%) but requires a moderately higher runtime (9.60 ms), reflecting the additional cost of non-linear kernel evaluation. Random Forest achieves competitive accuracy (96.18%) but is substantially slower, with an average runtime of 414.97 ms, due to the evaluation of multiple decision trees. In comparison, the LPTD baseline provides lower accuracy (88.74%) and a higher runtime than the fastest proposed classifiers (64.28 ms). These results indicate that the proposed classification-based predictors, particularly Voronoi and SVM-linear, offer a favourable accuracy–runtime trade-off for rapid topology determination in medium-resolution cryo-EM interpretation workflows.

## 3. Discussion

The experiments presented on the 38-protein benchmark indicate that formulating SSE correspondence as a supervised classification problem yields consistently high matching quality across diverse protein sizes, folds (α, β, and α/β), and map types (simulated and experimental). Across the full benchmark, all four classifiers operating under the unified pipeline (feature scaling where required, fixed hyperparameters, and identical train/test protocol) markedly outperform the prior LPTD baseline, with average correspondence accuracy clustering tightly around ~96% for SVM-RBF (95.99%), Random Forest (96.18%), and SVM-linear (96.22%), and peaking with Voronoi (96.82%). The narrow spread among the top three learning-based models suggests that the selected geometric feature space is highly informative and that the dominant performance gains arise from the classification formulation itself (i.e., learning discriminative decision rules), rather than from any single classifier family.

Deep-learning methods are highly valuable for map segmentation, SSE annotation, and backbone tracing, but they typically require larger labelled datasets, greater computational resources, and extensive tuning. By contrast, the proposed classification-based topology module is lightweight and interpretable, and it targets a distinct step: resolving SSE correspondence and directionality after the relevant SSEs have been detected. Thus, the proposed method can complement deep-learning-based cryo-EM workflows rather than directly competing with them.

The statistical analysis further supports this interpretation. Although Voronoi achieved the highest mean F1-score, its advantage over SVM-linear, SVM-RBF, and Random Forest was not statistically significant. Therefore, the primary contribution of the proposed framework lies in reformulating SSE correspondence as a classification-based learning problem that consistently improves performance over LPTD.

The expanded literature further clarifies the role of the proposed method relative to recent AI-based cryo-EM interpretation pipelines. Methods such as Haruspex, Emap2sec+, EMNUSS, cryoSSESeg, and DeepSSETracer focus mainly on density-map annotation or secondary-structure segmentation, whereas methods such as ModelAngelo, DeepMM, CR-I-TASSER, EMBuild, and DEMO-EM2 address broader automated model-building or assembly tasks. In contrast, the present framework targets a specific topology-resolution stage by determining correspondence and orientation between sequence/model-derived SSEs and density-map-derived SSEs. This distinction is important because incorrect SSE correspondence or reversed orientation can propagate into downstream topology, backbone tracing, and model-building errors even when secondary-structure regions are correctly detected.

A key observation is the systematic difference between helix and strand correspondence difficulty. Helix matching is highly stable across most proteins, with accuracies near the ceiling (often ≈ 0.97–1.00) for all evaluated supervised classifiers, and only a small subset of challenging cases exhibiting noticeable degradation (as reflected by the per-protein helix plot). In contrast, strand correspondence is visibly more variable and includes several proteins where accuracy drops substantially for all methods (as shown in the strand-only plot). This gap is expected in medium-resolution cryo-EM settings: helices typically manifest as long, tubular densities that are easier to segment into reliable sticks, whereas β-strands can be shorter, more tightly packed, and more sensitive to segmentation fragmentation or merging. Under the incomplete-matching evaluation protocol (matched vs. unmatched classes), such detection discrepancies translate directly into increased FP (spurious correspondences) and FN (missed true correspondences), which disproportionately affect strands.

Notably, despite the greater difficulty of strand matching, the supervised models still preserve strong relative performance. Voronoi tends to maintain the highest or near-highest strand accuracy across the set, which is consistent with its locally adaptive decision structure: it effectively partitions the feature space via proximity, without requiring global margin separation (SVM) or extensive ensemble aggregation (RF). SVM-linear remains highly competitive, implying that much of the separation between correct and incorrect correspondences is approximately linear in the chosen feature representation, while SVM-RBF provides only marginal gains in some proteins—suggesting limited benefit from introducing non-linear kernels once the features are well designed.

The length-dependent analysis further confirms that protein size influences correspondence difficulty. Smaller and medium-sized proteins generally yield near-perfect or very high F1-measure values, whereas larger proteins show a moderate performance reduction. This trend is expected because larger proteins often contain more secondary-structure elements, increasing the number of possible assignments and the likelihood of ambiguity among geometrically similar SSEs. However, the proposed supervised classifiers remain consistently more accurate than the LPTD baseline across length groups, indicating that the classification-based formulation improves robustness under increasing structural complexity.

From implementation perspective, the runtime results highlight a meaningful advantage of the proposed classification stage. The average runtime comparison shows that Voronoi is the fastest among the evaluated methods (on the order of only a few milliseconds per instance on average), followed by SVM-linear and SVM-RBF in the millisecond range. The Random Forest model is substantially slower than the other supervised classifiers, consistent with the overhead of evaluating multiple trees, while LPTD is also slower than the linear and nearest-neighbor classifiers in this setting. When compared with the runtime reported for LPTD in our earlier study, where the final three stages (vector construction, graph transformation, and similarity-based voting) required ~0.86 s for a small protein (1BZ4, 5 SSEs-A) and ~1.96 s for a large protein (5KBU, 65 SSEs-A), the proposed classification-based formulation is markedly more time-efficient in the correspondence prediction stage, completing in <1.2 s per protein on the same benchmark.

Although the benchmark includes both simulated and experimental cryo-EM maps, we acknowledge that further realism could be introduced into future simulated datasets by incorporating more advanced signal-degradation models. In this study, the simulated maps were intentionally retained in their original benchmark form to allow direct comparison with previously published topology-determination methods [1,3,38]. Nevertheless, future work could extend the simulation protocol by incorporating additional noise, anisotropic filtering, local-resolution variation, conformational heterogeneity, and map-processing artefacts. Such extensions would provide a more systematic assessment of robustness under increasingly realistic reconstruction conditions and would help further bridge the gap between simulated and experimental cryo-EM benchmarks.

In future work, we will extend the proposed classification-based correspondence and orientation framework to operate jointly across all chains of multi-chain (complex) proteins, enabling simultaneous, globally consistent topology recovery in assemblies rather than treating chains independently. We also plan to move beyond topology resolution and integrate an end-to-end structure reconstruction module that leverages the recovered SSE topology—together with the DTW-based direction assignment and geometric constraints—to generate an initial 3D backbone/trace hypothesis directly from medium-resolution cryo-EM maps. Finally, we will investigate richer feature representations and learning strategies (e.g., more expressive classifiers and cross-protein transfer settings) to improve robustness under severe segmentation errors, missing/extra sticks, and varying map resolutions, while maintaining the favourable runtime characteristics demonstrated in this study.

## 4. Materials and Methods

**Figure 11:**
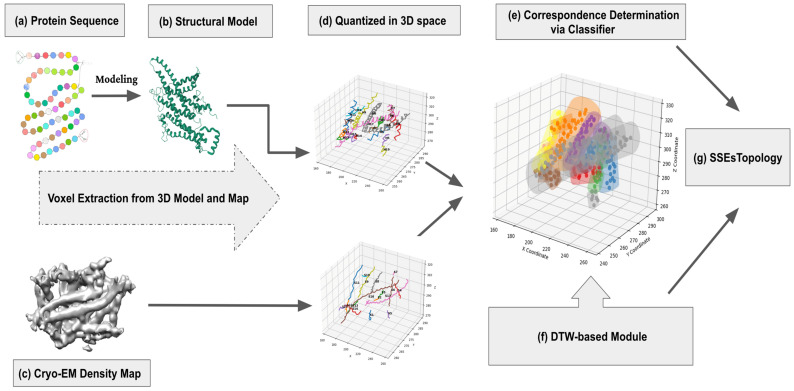
Overview of the proposed classification-based framework for secondary-structure topology determination from medium-resolution cryo-EM density maps (example: PDB 5I1M). (**a**) The protein sequence of 5I1M. (**b**) A 3D structural model. (**c**) The medium-resolution cryo-EM density map associated with 5I1M in 3D space. (**d**) Quantized in SSEs-S and SSEs-V in three-dimensional space and geometric cues are extracted from both SSEs-S and SSEs-V and organized into labelled samples in Cartesian coordinate space. (**e**) SSE correspondence is then formulated as a three-dimensional supervised classification problem. (**f**) A Dynamic Time Warping (DTW)-based module subsequently infers SSE orientation/directionality. The final output (**g**) is SSE topology in the cryo-EM map (correspondence + direction).

In this work, we introduce a fully automatic, classification-based computational framework for determining secondary-structure topology in medium-resolution cryo-EM density maps by establishing reliable correspondences between SSEs-S and SSEs-V. The method is designed to leverage the efficiency of classical supervised learning while remaining robust to the noise, heterogeneity, and limited resolvability that typically hinder topology recovery at 4–10 Å resolution. Rather than relying on density skeletonization or length-driven heuristics, we operate directly on 3D Cα coordinate sequences and map-derived SSE axes, enabling the classifier to learn intrinsic geometric characteristics from native spatial patterns. The key methodological contribution is to cast SSE correspondence as a three-dimensional classification problem in Cartesian space: both sources of SSE information are transformed into a common 3D grid representation, geometric cues are extracted, and candidate SSE pairings are encoded as labelled instances for supervised learning and ranking. We evaluate several complementary classifiers (SVM with linear/RBF kernels, Random Forest, and Voronoi) to obtain robust correspondence scores, and subsequently integrate a Dynamic Time Warping (DTW)-based orientation module to infer SSE directionality and resolve competing matches, yielding a unique and globally consistent SSE topology suitable for downstream model building and refinement. Figure 11 summarizes the proposed pipeline. Starting from the protein sequence (Figure 11a), an initial structural model is generated (Figure 11b) and used to extract sequence-derived secondary-structure elements as ordered Cα coordinate sequences for α-helices and β-strands. In parallel, the cryo-EM density map (Figure 11c) is processed with an SSE detection tool to identify map-derived SSEs as 3D stick/axis representations. To enable learning and matching in a unified space, both SSEs-S and SSEs-V are mapped into a common 3D Cartesian grid by discretizing their coordinates into voxels (Figure 11d) and computing geometric cues that capture intrinsic spatial patterns while avoiding reliance on density skeletons or length-based heuristics that can be unstable under noise and map heterogeneity. The correspondence problem is then reformulated as a supervised 3D classification task (Figure 11e): candidate SSE pairings are encoded as labelled training/test instances in Cartesian space and scored using robust distance-based ranking to prioritize plausible matches. We systematically evaluate multiple classifiers including SVM (linear and RBF kernels), Random Forest, and k-Nearest Neighbors (Voronoi) to identify reliable configurations for correspondence prediction. Finally, to obtain a unique and globally consistent topology, a Dynamic Time Warping (DTW)-based module (Figure 11f) estimates SSE orientation/directionality and resolves ambiguities among competing correspondences, yielding the final SSE topology in the cryo-EM map (Figure 11g), i.e., correspondence plus direction, which can be directly used for downstream model building and refinement.

### 4.1. Sequence-to-Structure Modelling

To embed sequence-level information into a three-dimensional representation suitable for geometric learning, we first generate a structural model of the target protein from its primary sequence. Following established topology-determination workflows, we adopt a comparative modelling strategy based on fold recognition and template-guided assembly. In practice, the sequence is submitted to a threading-based modelling pipeline (e.g., I-TASSER or an equivalent server), which searches the Protein Data Bank (PDB) for structurally related templates using meta-threading (e.g., LOMETS). The top-ranked alignments are combined to build an initial ensemble of candidate models and subsequently refined using replica-exchange Monte Carlo simulations or comparable conformational sampling. For each target, the final model is selected according to an internal confidence score that reflects both template reliability and convergence of the assembly process. Although AlphaFold can provide highly accurate predictions and could be used interchangeably within our downstream pipeline, we deliberately used I-TASSER here to match the modelling protocol adopted in prior related studies, enabling a direct, like-for-like comparison with previously reported correspondence methods.

From the selected model, each α-helix and β-strand is treated as a contiguous residue segment and assigned a unique SSE identifier. For every SSE, we extract the ordered backbone Cα coordinates, yielding a discrete 3D curve that captures its local geometry while remaining robust across modelling tools. Before learning, we apply standard preprocessing to ensure consistent representation and comparability with density-derived features: (i) coordinates are expressed in a common Cartesian reference frame to remove rigid-body offsets, (ii) each SSE curve is resampled to a fixed number of points via linear interpolation along the backbone path to normalize length while preserving shape, and (iii) auxiliary descriptors including SSE type (helix/strand), residue count, and a local axis vector are stored as associated metadata. These model-derived SSE representations form one side of the correspondence problem and are used as inputs to the supervised correspondence classifier.

### 4.2. Voxel Extraction from 3D Model and Cryo-EM Density Map

The second stage of the pipeline (Figure 11d) constructs compatible 3D Cartesian representations of secondary-structure elements from both the sequence and the cryo-EM map side, so that correspondence can be learned directly from geometry. On the model side, SSEs-S are already available as ordered Cα coordinate sequences extracted from the predicted atomic model (Section 4.1). These model-derived coordinate sequences provide an “ideal” geometric description of each SSE, independent of density skeletons or length-driven heuristics. On the cryo-EM side, the input is a volumetric density map in which SSEs at medium resolution appear as elongated rod-like (helices) or sheet-like (strands) regions rather than resolved atomic positions. We therefore apply an SSE detection tool (e.g., SSETracer [45], with SSEHunter as an alternative) to identify candidate helices/strands and estimate their locations and axes in 3D space. Each detected SSE is represented by a sequence of points (voxels) sampled along its central axis, forming a discrete curve that is geometrically analogous to the model Cα trace. To enable direct comparison, these density-derived voxel sequences are expressed in the same Cartesian reference frame adopted for the model. This shared voxel-based representation provides the common geometric language required to cast SSE correspondence as a 3D supervised classification problem in the subsequent stage and scored by the selected classifier to predict true correspondences.

### 4.3. Supervised Classification Formulation for SSE Correspondence

To establish correspondence between SSEs-S and SSEs-V, we cast the matching task as a supervised multi-class classification problem in 3D Cartesian space. Let $S={\left\{s_{i}\right\}}_{i=1}^{m}$ denotes the ordered set of model SSEs (represented by Cα-coordinate-derived geometric features) and $V={\left\{v_{j}\right\}}_{j=1}^{n}$ denotes the set of density-map sticks (represented by stick/axis-derived geometric features). Following the pipeline in Figure 11d,e, both (S) and (V) are first separated by SSE type into helix and strand subsets, {$S_{helix},S_{strand}$} and {$V_{helix},V_{strand}$} and two type-specific classifiers are trained to avoid cross-type confusion. In the training phase, each element $s_{i}\in S_{type}$ is assigned a unique class label ($L_{j}$), and a classifier $C_{type}$ is learned using supervised learning with hyperparameter optimization via grid search (minimizing validation error) to obtain ($\theta^{*}$) (e.g., (C,γ)) for SVM-RBF; (C) for linear SVM; {$N_{trees},maxdepth,minsplit$} for Random Forest). In the prediction phase, for each map stick $V_{j}\in V_{type}$, the corresponding trained classifier $C_{type}$ predicts a label $L_{pred}$, which directly identifies the matched model SSE ($s_{i}$) and forms a correspondence pair ($s_{i},v_{j}$). Repeating this procedure for all sticks yields the matching set (M), which serves as the estimated SSE correspondence between the model and the cryo-EM map. Algorithm 1 summarizes the proposed learning-based procedure for establishing SSE correspondence.
**Algorithm 1.** Learning-based SSE correspondence via supervised classification. The pseudocode outlines the training and inference workflow for mapping model-derived SSEs (SSEs-S) to map-derived SSEs (SSEs-V): S and V are first split by SSE type (helix/strand), type-specific classifiers are trained (with GridSearchCV hyperparameter tuning for SVM/RF or a distance metric for Voronoi), and each map $SSEv_{j}$ is classified to retrieve its matched model $SSEs_{i}$, yielding the correspondence set M.**Input:** Model SSE set S (SSEs-S), map SSE set V (SSEs-V), classifier type C **Output:** Correspondence set M**1.**$Split\;S\;and\;V\;by\;SSEtype:\;\{S_{helix},S_{strand}\},\{V_{helix},V_{strand}\}.$**2.***For each* t∈{helix,strand} *do***3.**             *Construct labelled training set* $D_{t}$ *from* $S_{t}$ *(each *
$s_{i}\in S_{t}$ *assigned label *
$L_{i}$
*).*
**4.**             *If*
$\;C\in \left\{SVM-linear,SVM-RBF,RF\right\}\;$
*then*
**5.**             $\theta_{t}\leftarrow GridSearchCV\left(D_{t},C\right)$
**6.**             $Else\left(C=kNN\left(1-NN\right)\right)$
**7.**                       *Set distance metric (e.g., Euclidean);*
$\;\theta_{t}\leftarrow \emptyset$
**8.**             *End if*
**9.**             *Train classifier* $C_{t}\leftarrow Train\left(D_{t},C,\theta_{t}\right)$**10.***End for***11.***Initialize* M←∅.**12.***For each map-derived SSE* $v_{j}\in V$ *do***13.**             *Let* t← *type of* $v_{j}$ *(helix/strand); select classifier *
$C_{t}$
**14.**             $L_{pred}\leftarrow Predict\left(C_{t},v_{j}\right)$
**15.**             *Retrieve* $s_{i}$*∈* $S_{t}$ *with label*
$L_{pred};$ *add (*$s_{i},$$v_{j}$*) to M.***16.***End for***17.***Return M.*

Hyperparameters were selected via cross-validated tuning on the training set. For SVM-linear, we tuned the regularization parameter C. For SVM-RBF, we optimized both C and the kernel width γ. For Random Forest, we tuned the number of trees, maximum tree depth, and the minimum number of samples required to split an internal node.

To robustly quantify correspondence performance under different decision boundaries and noise regimes, we evaluated four supervised classifiers: SVM with a linear kernel, SVM with an RBF kernel, Random Forest (RF), and k-Nearest Neighbors (Voronoi). Linear SVM provides a strong low-variance baseline when the learned feature space is approximately separable, whereas RBF-SVM captures non-linear class boundaries that may arise from complex SSE geometries. RF was included for its ensemble robustness and reduced sensitivity to feature scaling, and Voronoi was used as a parameter-free proximity baseline driven solely by the chosen distance metric (Euclidean). For all methods, hyperparameters were selected using a benchmark-level grid search procedure rather than a conventional k-fold cross-validation scheme. For each classifier, candidate hyperparameter configurations were evaluated across the full benchmark, and the configuration yielding the highest average F1-score was selected. The selected hyperparameters were then fixed and applied consistently across all 38 benchmark proteins to ensure a fair, like-for-like comparison among predictors. Specifically, we tuned C for linear SVM; C,γ for RBF-SVM; and RF settings such as number of trees, maximum depth, and minimum samples per split. Feature scaling was applied where appropriate to prevent distance-based and margin-based classifiers from being dominated by high-magnitude feature dimensions. The optimal hyperparameter configuration was then fixed and applied uniformly across all benchmark proteins to enable a fair comparison across datasets and SSE types. The predicted correspondence scores were subsequently propagated to the downstream modules (including orientation resolution) to yield a globally consistent topology solution.

### 4.4. Orientation (Direction) Resolution Using DTW

After the correspondence module assigns each map-derived density stick $v=\left[q_{1},\ldots ,q_{n}\right]\;$ to a specific model-derived SSE $s=\left[p_{1},\ldots ,p_{m}\right]$ (Figure 11e), we resolve the remaining ambiguity in directionality using a Dynamic Time Warping (DTW)–based algorithm (Figure 11f). DTW provides a robust similarity metric between two discrete 3D curves by computing an optimal non-linear alignment that minimizes the cumulative Euclidean distance along a dynamic-programming cost matrix *D*, where each entry aggregates the local point-to-point cost with the minimum of insertion, deletion, or match transitions (Algorithm 2, lines 1–11). For each matched pair, we compute the DTW cost in the forward orientation, ${dist}_{forward}=DTW\left(s,v\right)$ and in the reverse orientation, ${dist}_{backward}=DTW$ (Algorithm 2, lines 12–14). The stick direction is then determined by comparing these two costs: the direction is set $d=+1\left(forward\right)\;$ if ${dist}_{forward}<{dist}_{backward}$; otherwise, it is set to $d=-1\left(reverse\right)$ (Algorithm 2, lines 15–16).
**Algorithm 2.** Direction detection using DTW**Input**: $s=\left[p_{1},\ldots ,p_{m}\right]$ (model SSE coordinates), $v=\left[q_{1},\ldots ,q_{n}\right]$ (map stick coordinates) **Output:**
d∈{+1,−1} (orientation; +1 forward, −1 reverse)**1.**$FunctionDTW\left(SeqA,SeqB\right)$**2.**       $m\leftarrow \left|seqA\right|,n\leftarrow \left|seqB\right|$
**3.**       $InitializeD\in R^{\left(m+1\right)\times \left(n+1\right)}with+\infty ;setD\left[0,0\right]\leftarrow 0$
**4.**       For i=1 to m:
**5.**             For j=1 to n:
**6.**                    $cost\leftarrow {\left|\left|seqA[j]-SeqB\left[j\right]\right|\right|}_{2}$
**7.**                    $D\left[i,j\right]\leftarrow cost+min\{D\left[i-1,j\right],D\left[i,j-1\right],D\left[i-1,j-1\right]\}$
**8.**             EndFor
**9.**       EndFor
**10.**       $ReturnD\left[m,n\right]$
**11.**EndFunction**12.**${dist}_{F}\leftarrow DTW\left(s,v\right)$**13.**$v^{rev}\leftarrow Reverse\left(v\right)$**14.**${dist}_{B}\leftarrow DTW\left(s,v^{rev}\right)$**15.**$If{dist}_{F}<{dist}_{B}thend\leftarrow +1elsed\leftarrow -1$**16.**Returnd

## 5. Conclusions

Secondary-structure topology determination is a pivotal intermediate step for interpreting medium-resolution cryo-EM density maps, where individual residues are not reliably resolved and model construction must rely on correctly identified and connected helices and strands. In this resolution, accurate SSE correspondence and orientation assignment provide the structural constraints needed to place and trace secondary-structure elements consistently within the cryo-EM map, thereby stabilizing subsequent backbone construction, reducing topological ambiguities, and improving the reliability of downstream model building and refinement. In this study, we proposed and validated an automated correspondence-learning framework for resolving SSE correspondence in medium-resolution cryo-EM density maps, with the specific goal of producing accurate and stable SSE-to-stick matchings that enable robust topology reconstruction. Using an integrated benchmark of 38 proteins assembled from previous studies [1,3,38] spanning broad structural complexity and including both simulated and experimental maps, the proposed supervised formulation demonstrated strong robustness under incomplete matching and segmentation uncertainty. Across the benchmark, all four evaluated supervised predictors substantially outperformed the prior LPTD baseline, achieving ~96% average correspondence accuracy, with Voronoi providing the best overall mean performance (96.82%) and SVM-linear delivering nearly identical accuracy (96.22%) with very low runtime. The results further show that helix correspondence can be recovered with near-ceiling accuracy for most targets, while strand correspondence remains more challenging yet still benefits strongly from supervised learning. Finally, by coupling correspondence prediction with DTW-based orientation detection, the pipeline produces directionally consistent pairings required for subsequent topology assembly, reducing the risk of reversed sequence tracing. Overall, the proposed method offers a practical combination of accuracy, stability, and computational efficiency, providing a strong basis for future extensions of cryo-EM interpretation pipelines while reducing downstream ambiguity during model building.

## Figures and Tables

**Table 1:** Benchmark proteins and secondary-structure statistics used in this study. For each target protein, the table reports the PDB identifier (with the corresponding EMDB accession when available), the number of amino acids, protein class (α, β, or α/β), the number of model-derived α-helices $N_{\propto }$, and β-strands $N_{\beta }$, the total number of model SSEs $N_{\propto +\beta }$, and the number of map-derived density SSEs (SSEs_V) detected for helices and strands ($N_{{Sticks}_{H}}$ and $N_{{Sticks}_{S}}$, respectively).

Row	PDB ID (EMDB ID)	AA Count	∝/∝-β Protein	$N_{\propto }$	$N_{\beta }$	$N_{\propto +\beta }$	$N_{{Sticks}_{H}}$	$N_{{Sticks}_{S}}$
1	1A7D	118	∝ protein	6	-	6	4	-
2	1BZ4	144	∝ protein	5	-	5	5	-
3	1FLP	142	∝ protein	7	-	7	6	-
4	1HG5	289	∝ protein	11	-	11	9	-
5	1HZ4	373	∝ protein	21	-	21	19	-
6	1ICX	155	∝-β protein	6	7	13	4	7
7	1LWB	122	∝ protein	6	-	9	6	-
8	1NG6	148	∝ protein	9	-	6	7	-
9	1OZ9	150	∝-β protein	5	5	10	5	4
10	1P5X	245	∝ protein	19	-	19	13	-
11	1XQO	256	∝ protein	14	-	14	14	-
12	1YD0	96	∝-β protein	5	3	8	4	3
13	1Z1L	345	∝ protein	23	-	23	14	-
14	2OVJ	201	∝ protein	12	-	12	8	-
15	2XB5	207	∝ protein	13	-	13	9	-
16	2XVV	585	∝ protein	33	-	33	19	-
17	2Y4Z	140	∝-β protein	6	2	8	6	2
18	3ACW	293	∝ protein	17	-	17	14	-
19	3HJL	329	∝ protein	20	-	20	20	-
20	3IEE	270	∝ protein	9	-	9	8	-
21	3IXV	222	∝ protein	14	-	14	10	-
22	3LTJ	201	∝ protein	16	-	16	12	-
23	3ODS	415	∝ protein	21	-	21	16	-
24	4OXW	119	∝-β protein	5	3	8	4	3
25	4R9A	144	β protein	-	11	11	22	10
26	4UE4	102	∝ protein	6	-	6	5	-
27	4YOK	204	β protein	16	-	16	14	-
28	3C91 (EMD-1733)	233	∝-β protein	8	10	18	6	9
29	3FIN (EMD-5030)	117	∝ protein	4	-	4	4	-
30	4CHV (EMD-2526)	361	∝-β protein	15	8	23	15	7
31	5I1M (EMD-8070)	458	∝ protein	19	-	19	17	-
32	5KBU (EMD-8231)	1034	∝-β protein	33	32	65	28	26
33	5M50 (EMD-4154)	439	∝-β protein	15	12	27	14	12
34	5O8O (EMD-3761)	349	∝-β protein	3	21	24	3	19
35	5UZB (EMD-8625)	177	∝-β protein	9	4	13	6	3
36	6EM3 (EMD-3888)	291	∝-β protein	3	8	11	3	6
37	6F36 (EMD-4176)	327	∝ protein	13	-	13	11	-
38	6UXW (EMD20934)	1703	∝-β protein	33	10	43	27	8

**Table 2:** Statistical significance analysis of pairwise classifier comparisons using paired *t*-tests on per-protein F1-scores across the 38-protein benchmark. A *p*-value below 0.05 was considered statistically significant.

Method A	Method B	*p*-Value	Significant
Voronoi	SVM-linear	0.109	No
Voronoi	Random Forest	0.241	No
Voronoi	SVM-RBF	0.183	No
Voronoi	LPTD	0.000	Yes
SVM-linear	Random Forest	0.943	No
SVM-linear	SVM-RBF	0.695	No
SVM-linear	LPTD	0.001	Yes
Random Forest	SVM-RBF	0.601	No
Random Forest	LPTD	0.001	Yes
SVM-RBF	LPTD	0.002	Yes

Note: Significance was determined at *p* < 0.05. Non-significant comparisons indicate that differences among the proposed supervised classifiers are not statistically distinguishable, whereas significant comparisons with LPTD indicate that the proposed classification-based methods outperform the baseline.

## Data Availability

To promote transparency, reproducibility, and reuse, the full implementation of the proposed framework (source code, benchmark data, and supporting scripts) is available at https://github.com/parsa1601/cryotopo-ml (accessed on 6 May 2026). These materials are sufficient to reproduce the experiments and regenerate the figures reported in this study.

## References

[1] Behkamal B., Naghibzadeh M., Pagnani A., Saberi M.R., Al Nasr K. (2021). Solving the α-helix correspondence problem at medium-resolution Cryo-EM maps through modeling and 3D matching. J. Mol. Graph. Model..

[2] Jamali K., Käll L., Zhang R., Brown A., Kimanius D., Scheres S.H.W. (2024). Automated model building and protein identification in cryo-EM maps. Nature.

[3] Behkamal B., Naghibzadeh M., Pagnani A., Saberi M.R., Al Nasr K. (2022). LPTD: A novel linear programming-based topology determination method for cryo-EM maps. Bioinformatics.

[4] Bepler T., Morin A., Rapp M., Brasch J., Shapiro L., Noble A.J., Berger B. (2019). Positive-unlabeled convolutional neural networks for particle picking in cryo-electron micrographs. Nat. Methods.

[5] Al-Haija Q., Al Nasr K. Supervised Regression Study for Electron Microscopy Data. Proceedings of the 2019 IEEE International Conference on Bioinformatics and Biomedicine (BIBM).

[6] Chiu W., Schmid M.F. (1997). Pushing back the limits of electron cryomicroscopy. Nat. Struct. Biol..

[7] Zhou Z.H., Dougherty M., Jakana J., He J., Rixon F.J., Chiu W. (2000). Seeing the herpesvirus capsid at 8.5 A. Science.

[8] Walls A.C., Park Y.-J., Tortorici M.A., Wall A., McGuire A.T., Veesler D. (2020). Structure, Function, and Antigenicity of the SARS-CoV-2 Spike Glycoprotein. Cell.

[9] Yan R., Zhang Y., Li Y., Xia L., Guo Y., Zhou Q. (2020). Structural basis for the recognition of SARS-CoV-2 by full-length human ACE2. Science.

[10] Nakamura T., Wang X., Terashi G., Kihara D. (2023). DAQ-Score Database: Assessment of map–model compatibility for protein structure models from cryo-EM maps. Nat. Methods.

[11] DeVore K., Chiu P.-L. (2022). Probing Structural Perturbation of Biomolecules by Extracting Cryo-EM Data Heterogeneity. Biomolecules.

[12] Al Nasr K., He J. (2016). Constrained cyclic coordinate descent for cryo-EM images at medium resolutions: Beyond the protein loop closure problem. Robotica.

[13] Al Nasr K., He J. (2009). An effective convergence independent loop closure method using Forward-Backward Cyclic Coordinate Descent. Int. J. Data Min. Bioinform..

[14] Casañal A., Shakeel S., Passmore L.A. (2019). Interpretation of medium resolution cryoEM maps of multi-protein complexes. Curr. Opin. Struct. Biol..

[15] Alshammari M., Wriggers W., Sun J., He J. (2022). Refinement of AlphaFold2 models against experimental and hybrid cryo-EM density maps. QRB Discov..

[16] Kleywegt G.J., Adams P.D., Butcher S.J., Lawson C.L., Rohou A., Rosenthal P.B., Subramaniam S., Topf M., Abbott S., Baldwin P.R. (2024). Community recommendations on cryoEM data archiving and validation. IUCrJ.

[17] Bendory T., Bartesaghi A., Singer A. (2020). Single-particle cryo-electron microscopy: Mathematical theory, computational challenges, and opportunities. IEEE Signal Process. Mag..

[18] Levy A., Poitevin F., Martel J., Nashed Y., Peck A., Miolane N., Ratner D., Dunne M., Wetzstein G. (2022). CryoAI: Amortized Inference of Poses for Ab Initio Reconstruction of 3D Molecular Volumes from Real Cryo-EM Images. Proceedings of the Computer Vision—ECCV 2022: 17th European Conference, Tel Aviv, Israel, 23–27 October 2022.

[19] Kim H.-U., An M.Y., Kim Y.K., Chung J.M., Jung H.S. (2025). Combining Cryo-EM with Computational Approaches To Revolutionize Structural Biology. Protein J..

[20] Bouvier G., Bardiaux B., Pellarin R., Rapisarda C., Nilges M. (2022). Building Protein Atomic Models from Cryo-EM Density Maps and Residue Co-Evolution. Biomolecules.

[21] Iqbal S., Eng E.T., Kamal M.A., Shen B. (2026). Artificial intelligence in cryo-EM: Emerging deep neural network methods from sample preparation, particle picking, map reconstruction, modelling to enhanced resolution. BMC Artif. Intell..

[22] Aishima J., Russel D.S., Guibas L.J., Adams P.D., Brunger A.T. (2005). Automated crystallographic ligand building using the medial axis transform of an electron-density isosurface. Acta Crystallogr. Sect. D.

[23] Si D., Ji S., Al Nasr K., He J. (2012). A machine learning approach for the identification of protein secondary structure elements from cryoEM density maps. Biopolymers.

[24] Bansia H., des Georges A. (2025). Connecting the dots: Deep learning-based automated model building methods in cryo-EM. Front. Mol. Biosci..

[25] Si D., Nakamura A., Tang R., Guan H., Hou J., Firozi A., Cao R., Hippe K., Zhao M. (2022). Artificial intelligence advances for de novo molecular structure modeling in cryo-electron microscopy. Wiley Interdiscip. Rev. Comput. Mol. Sci..

[26] Sanchez-Garcia R., Gomez-Blanco J., Cuervo A., Carazo J.M., Sorzano C.O.S., Vargas J. (2021). DeepEMhancer: A deep learning solution for cryo-EM volume post-processing. Commun. Biol..

[27] Li R., Si D., Zeng T., Ji S., He J. (2016). Deep convolutional neural networks for detecting secondary structures in protein density maps from cryo-electron microscopy. 2016 IEEE International Conference on Bioinformatics and Biomedicine (BIBM).

[28] Bataineh M., Al Nasr K., Mu R., Alamri M., Peng W., Cai Z., Skums P. (2024). Correction to: Deep Learning Approach to Identify Protein’s Secondary Structure Elements. Bioinformatics Research and Applications (ISBRA 2024).

[29] He J., Huang S.-Y. (2021). EMNUSS: A deep learning framework for secondary structure annotation in cryo-EM maps. Brief. Bioinform..

[30] Sazzed S. (2024). Determining Protein Secondary Structures in Heterogeneous Medium-Resolution Cryo-EM Images Using CryoSSESeg. ACS Omega.

[31] Jumper J., Evans R., Pritzel A., Green T., Figurnov M., Ronneberger O., Tunyasuvunakool K., Bates R., Žídek A., Potapenko A. (2021). Highly accurate protein structure prediction with AlphaFold. Nature.

[32] LeCun Y., Bengio Y., Hinton G. (2015). Deep learning. Nature.

[33] Samek W., Wiegand T., Müller K.-R. (2017). Explainable Artificial Intelligence: Understanding, Visualizing and Interpreting Deep Learning Models. arXiv.

[34] Shorten C., Khoshgoftaar T.M. (2019). A survey on Image Data Augmentation for Deep Learning. J. Big Data.

[35] Si D., Moritz S.A., Pfab J., Hou J., Cao R., Wang L., Wu T., Cheng J. (2020). Deep Learning to Predict Protein Backbone Structure from High-Resolution Cryo-EM Density Maps. Sci. Rep..

[36] Al Nasr K., Ranjan D., Zubair M., Chen L., He J. (2014). Solving the Secondary Structure Matching Problem in Cryo-EM De Novo Modeling Using a Constrained K-Shortest Path Graph Algorithm. IEEE/ACM Trans. Comput. Biol. Bioinform..

[37] Singer A., Sigworth F.J. (2020). Computational Methods for Single-Particle Electron Cryomicroscopy. Annu. Rev. Biomed. Data Sci..

[38] Behkamal B., Naghibzadeh M., Saberi M.R., Tehranizadeh Z.A., Pagnani A., Al Nasr K. (2021). Three-Dimensional Graph Matching to Identify Secondary Structure Correspondence of Medium-Resolution Cryo-EM Density Maps. Biomolecules.

[39] Al Nasr K., Ranjan D., Zubair M., He J. (2011). Ranking Valid Topologies of the Secondary Structure elements Using a constraint Graph. J. Bioinform. Comput. Biol..

[40] Al Nasr K., Yousef F., Jebril R., Jones C. (2018). Analytical Approaches to Improve Accuracy in Solving the Protein Topology Problem. Molecules.

[41] Abeysinghe S., Ju T., Baker M.L., Chiu W. (2008). Shape modeling and matching in identifying 3D protein structures. Comput.-Aided Des..

[42] Baker M.L., Ju T., Chiu W. (2007). Identification of secondary structure elements in intermediate-resolution density maps. Structure.

[43] He J., Huang S.-Y. (2021). Full-length de novo protein structure determination from cryo-EM maps using deep learning. Bioinformatics.

[44] Zhang X., Zhang B., Freddolino L., Zhang Y. (2022). CR-I-TASSER: Assemble protein structures from cryo-EM density maps using deep convolutional neural networks. Nat. Methods.

[45] Si D., He J. (2013). Beta-sheet Detection and Representation from Medium Resolution Cryo-EM Density Maps. BCB’13: Proceedings of the International Conference on Bioinformatics, Computational Biology and Biomedical Informatics.

[46] Mostosi P., Schindelin H., Kollmannsberger P., Thorn A. (2020). Haruspex: A Neural Network for the Automatic Identification of Oligonucleotides and Protein Secondary Structure in Cryo-Electron Microscopy Maps. Angew. Chem. Int. Ed..

[47] Wang X., Alnabati E., Aderinwale T.W., Maddhuri Venkata Subramaniya S.R., Terashi G., Kihara D. (2021). Detecting protein and DNA/RNA structures in cryo-EM maps of intermediate resolution using deep learning. Nat. Commun..

[48] Mu Y., Sazzed S., Alshammari M., Sun J., He J. (2021). A Tool for Segmentation of Secondary Structures in 3D Cryo-EM Density Map Components Using Deep Convolutional Neural Networks. Front. Bioinform..

[49] He J., Lin P., Chen J., Cao H., Huang S.-Y. (2022). Model building of protein complexes from intermediate-resolution cryo-EM maps with deep learning-guided automatic assembly. Nat. Commun..

[50] Zhang Z., Cai Y., Zhang B., Zheng W., Freddolino L., Zhang G., Zhou X. (2024). DEMO-EM2: Assembling protein complex structures from cryo-EM maps through intertwined chain and domain fitting. Brief. Bioinform..

[51] Giri N., Wang L., Cheng J. (2024). Cryo2StructData: A Large Labeled Cryo-EM Density Map Dataset for AI-based Modeling of Protein Structures. Sci. Data.

